# Supplementary material for: Patterns of Intron Gain and Loss in Fungi
Source: PLoS Biol. 2004 Nov 30;2(12):e422. doi: 10.1371/journal.pbio.0020422 (PMC532390; doi:10.1371/journal.pbio.0020422)
Supplement: Table S1 — Also available at http://genes.mit.edu/NielsenEtAl/. (4.3 MB ZIP). [file pbio.0020422.st001.zip › NielsenEtAl/html/1287.html]

AN2555.1.NCU06720.1.MG09538.1.FG05797.1


```
 CLUSTAL W (1.82) Multiple Sequence Alignments - Introns Inserted


Sequence 1: NCU06720.1	577 aa
Sequence 2: MG09538.1	604 aa
Sequence 3: AN2555.1	541 aa
Sequence 4: FG05797.1	568 aa
Alignment Length: 625 aa
Number Identitical Residues: 150 aa
Alignment Score (without introns) 9130


MG09538.1 	MHFQLSASAWLLLLLGEATGLRSLQHVGKRDPVWIQSAPLQKYEVSIDSTEDVNTTATET
NCU06720.1	MRLTALVSSLLLATTATAGGP-SFGQAEKKYLEHVANR-LKNHAAKVEAAPAVER-----
FG05797.1 	MRFSLITSSFLALGSFVEAGF---NQGAIRAFDHPHPRGYDQKRAPVPPKPAFEK-----
AN2555.1  	MGVGNVLYAAAVAVSLCTDYVAASSPHGRFGQRARDSVNLAKRASHLHNATASGK-----
          	* .     :             :                  :    :             

MG09538.1 	HAVVRRAQPLFLTDKTRK1FSVDGNGIPEVKFDVGESYAGQLPISNNSTDENKMFFWFFP
NCU06720.1	-DVAKVQEPKFLNANTTR1FAVDGKNIPDVDFDVGESYAGTLPISQDPEEKSELFWWFFP
FG05797.1 	-----RAKSKFLNKKSEK~FAVNGSAIPEVDFDVGESYAGLLPISQSPDEERELFFWFFP
AN2555.1  	---LPKSDFRFLTDKTSP1FLVD--HLPDVHFDVGELYSGLIPIEKG-NDSRALFFAFQP
          	       .  **. ::   * *:   :*:*.***** *:* :**.:.  :.  :*: * *

MG09538.1 	SQNPLAKKEIMIW~LNGG0PGCSSMAGLMQENGPFLWQSGTFRPVKNPFSWTEL~TNVVW
NCU06720.1	STNPAAKKEILIW~LNGG0PGCSSLEGFLQENGPFLWQYGTYKPVKNPWSWHTL~TNVIW
FG05797.1 	STNPDAGEEVLIW2LNGG~PGCSSLSGLLTENGPFLWQQGTLAPVANSYSWTNL2TNVIW
AN2555.1  	KLG-DPVDEVTIW~LNGG~PGCSSLEGFFQENGRFLWLPGTYQPVENPYSWVNL~TNVLW
          	. .  . .*: ** **** *****: *:: *** ***  **  ** *.:**  * ***:*

MG09538.1 	~IEQPVGTGFATGKPNATNEEDVARQFLGFWRNFVDTFALQGYKVYITGESYAGAYCPYI
NCU06720.1	~VEQPVGTGFSTGNVTATSEEDVAAQFMGFWKNFIDTFAMQGYKVYITGESYAGLYCPYI
FG05797.1 	~IEQPVGVGYSQGKPNITNEVELGLQFIGFWRNFIDTFDLKGATTYITGESYAGYYVPYI
AN2555.1  	2VDQPVGTGFSVGKPTAISQEETAEDFVKFFKNFQQTFGIKNFKIYVTGESYAGRYVPYV
          	 ::****.*:: *: .  .: : . :*: *::** :** ::. . *:******* * **:

MG09538.1 	ADAMIKANDTQYFNMTG~MLIYDPVIGE-DSMQEEVPVMGLINAWPGLFPFNETFRADLQ
NCU06720.1	ASAFLDANDKTYYDMSG~LMIYDPVISW-DEVAEQVPVVQFTEYWSGLFPFNDTFREHIK
FG05797.1 	ADAFITAADDEYYKLGG~VAINDPLLGD-GTLQQQAVIMPFVEYWDKLFYLNETTMNALR
AN2555.1  	SAAMLDQNDTEYYDLAG1ALVYDPCIGQHDVVQEQIPAVPFVQANANLFNFNSSFMAQIE
          	: *::   *  *:.: *   : ** :.  . : ::   : : :    ** :*.:    :.

MG09538.1 	ARDAKCGLTEFMTNGLSYPPKGPLSSVSP-VEKD----EKCVNIFDDVYKAAFEINPCFN
NCU06720.1	SVDAKCGYSDFVSQYLVYPPKGLQPSKLPGTDKTGTTRDECWGLFYEIFDAILLLNPCFN
FG05797.1 	WTHEHCGFDKYLEKYGTFPPPEGKFPVLPDPYKDT--KNYTCDMFDLVYASALDSNPCFN
AN2555.1  	EMHQTCGYADFIDQYLVFPPSGVQPPKYFNYSSEA---DLACDVFDLINNEAFAANTCFD
          	  .  **  .:: :   :**     .   .  .     :   .:*  :    :  *.**:

MG09538.1 	IYEITQSCPLLWDVLGF1PTS~LDY1VPEGA-TIYFNRTDVKRAIHAPENVNWEVCKDG-
NCU06720.1	IYQVATTCPLLWDVLGF~PGS~MGY~LPEGA-KVYFDRDDVKKAVHAPENVKWSSCSEQN
FG05797.1 	IYHITDTCPKTYSQLGI~VNQ0NDY~SPPGA-EVYFNRTDVKKALHAPLDSIWYQCTPNN
AN2555.1  	IYEVNLMCPLQWDVLAF~PTE~LTY~QPDGAPTVYFDRPDVKRAMHAPQSVEWSICSNHA
          	**.:   **  :. *.:   .   *  * **. :**:* ***:*:*** .  *  *.   

MG09538.1 	IFKNG-------TDRSPPSSRAALPNVIDHTQNVIIGHGALDMVLIANGTLLSIQNMTWG
NCU06720.1	VFVGG-------RDRSEPSSFHALPHVIDATKNVIIGHGALDMVLIANGTLLGIQNMTWG
FG05797.1 	VFGFGNPNSTRSDKSLAPAQDGVLKRVIEHTNNTIIGVGRLDFILPPNGTLFALQNVTWN
AN2555.1  	VFMGGDAGPESEGDISANPIEHVLPQVIEATNRVLIGNGDYDMIIITNGTLLSIQNMTWN
          	:*  *....  . .    .   .* .**: *:..:** *  *::: .****:.:**:**.

MG09538.1 	GKLGFQSRP-SAPFFVPRHR-----RDFKSKQGLGTLTGSGVMGFTHTERGLTYVGVEMS
NCU06720.1	GKLGFQNKP-DQPFYVP----------YNTMSDLSTLAAGGVFGSMGSERGLTWVQIDMS
FG05797.1 	GQQGYQKYPQDKQFYVP----------FHPEYNGGRLSEAGIVGQWGYERGLTYYEVQLA
AN2555.1  	GKLGFQERP-SEPIYLGADLPDLEWAEVRAENGLDVNQPQGTMGVQHYERGLMWAETYQS
          	*: *:*. * .  :::    .. .  . ..  . .     * .*    **** :     :

MG09538.1 	GHMVPQYQPAAAFRHVEVLLGRVANLSSTQPFTTQMNYTQPKPSELGGPPATANSPVGGG
NCU06720.1	GHMVPQYAPSAAYRQLEFLLGRVNCLNCTVPFTTDPDAPQ------------SQQPLGEG
FG05797.1 	GHELPGYSAGAGYRVVEALLGRIKNLGVVEDFTTQK------------------GDFQGN
AN2555.1  	GHMTPEYQPRVAYRHLEWLLGRVEKL----------------------------------
          	**  * * . ..:* :* ****:  *                                  

MG09538.1 	SGRGSPKSESPRLSSPSTLLPLAILVPLGFALVS
NCU06720.1	TAPQS-WSQSPGKTGKQHGGGRGRFVR-------
FG05797.1 	ATAMSMKGLNPLGQPFGHGPVY------------
AN2555.1  	----------------------------------
          	
```
